# Determination of ED_50_ and ED_95_ of remimazolam besylate combined with alfentanil for adult gastroscopy: a prospective dose-finding study

**DOI:** 10.1016/j.bjane.2024.844518

**Published:** 2024-05-23

**Authors:** Pingjuan Wang, Song Xue, Liufei Zhang, Kunkun Gao, Yiqiao Wang

**Affiliations:** The Fifth Clinical Medical College of Anhui Medical University, Anhui nº 2 Provincial People's Hospital, Hefei, Anhui, China

**Keywords:** Alfentanil, Dose, Gastroscopy, Remimazolam besylate, Sequential method

## Abstract

**Background:**

To explore the median effective dose (ED50) and 95% effective dose (ED95) of remimazolam besylate combined with alfentanil for adult gastroscopy.

**Methods:**

This prospective studyenrolled 31 patients scheduled to painless gastroscopy at Anhui No. 2 Provincial People's Hospital between April and May, 2022. 5 µg.kg^−1^ of alfentanil hydrochloride was used for pre-analgesia. The initial single loading dose of remimazolam besylate was 0.12 mg.kg^−1^, increased or reduced by 0.01 mg.kg^−1^ for the next patient with modified Dixon sequential method. The modified Observer's Assessment of Alertness/Sedation Scale (MOAA/S) was used to assess sedation.

**Results:**

Combined with alfentanil, the ED50 of remimazolam besylate was 0.147 mg.kg^−1^ (95% CI: 0.138-0.160 mg.kg^−1^) and ED95 0.171 mg.kg^−1^ (95% CI: 0.159-0.245 mg.kg^−1^). The induction time after injection of remimazolam besylate was 70 ± 25 s, with the anesthesia recovery time and the observation time in resuscitation room 5.13 ± 2.13 min and 2.32 ± 1.6 min, respectively. Twenty nine patients’ vital signs were within acceptable limits during gastroscopy.

**Conclusions:**

The ED50 of remimazolam besylate combined with alfentanil for painless gastroscopy was 0.147 mg.kg^−1^, and the ED95 was 0.171 mg.kg^−1^.

## Introduction

An increasing number of patients currently undergo gastroscopy under anesthesia, providing conditions for a thorough examination. It has been reported that painless gastroscopy has a much lower incidence of intraoperative and postoperative adverse reactions like nausea and vomiting, minimizing the risk of physical injury during examination.

Remimazolam besylate is a new type of water-soluble ultra-short-acting benzodiazepine for sedation and anesthesia.^1^ Previous studies have suggested that it can safely and quickly induce sleep in patients undergoing painless colonoscopy and shorten anesthesia induction time. It also avoids adverse events like low Saturation of Peripheral Oxygen (SpO_2_) and injection pain.^2^ Due to its late application, phase III clinical studies were conducted mainly in painless colonoscopy, with a recommended loading dose of 7.5 mg and an additional dose of 2.5 mg.^1^ However, this did not achieve effective sedation in painless gastroscopy, with a majority of patients exhibiting failure of sedation, significant body movement, and choking and coughing when the endoscope was inserted.^3^^,^^4^ Alfentanil is a short-acting opioid widely used for intraoperative analgesia, including endoscopy.^5^ The effective dose of remimazolam besylate combined with alfentanil in painless gastroscopy remains unknown.

Herein, we aimed to explore the ED_50_ and ED_95_ of the initial loading of remimazolam besylate combined with alfentanil for adult painless gastroscopy.

## Methods

### Study design and participants

This study was designed with up-and-down sequential allocation, enrolling patients scheduled to undergo painless gastroscopy at Anhui nº 2 Provincial People's Hospital between April and May 2022. The study was approved by the Ethics Committee of Anhui nº 2 Provincial People's Hospital and registered in the Chinese Clinical Trial Registry (ChiCTR2200060354). All patients voluntarily signed the informed consent.

### Interventions

Patients were fasted for 6–8 hours and abstained from water for 2–4 hours before anesthesia. All drugs were avoided. Heart Rate (HR), Systolic Bblood Pressure (SBP), Mean Arterial Pressure (MAP) and SPO_2_ were routinely monitored. In the left decubitus position, patients received a slow intravenous injection of alfentanil hydrochloride of 5 μg.kg^−1^ (Yichang Humanwell Pharmaceutical Co., Ltd., 50 μg.mL^−1^ in saline) for analgesic pretreatment. About one minute later, remimazolam besylate (Yichang Humanwell Pharmaceutical Co., Ltd., 25 mg by C_21_H_19_BrN_4_O_2_) was single-injected. As reported, the initial loading dose of remimazolam besylate was set at 0.12 mg.kg^−1^, which was increased or reduced by 0.01 mg.kg^−1^ for the next patient with the modified Dixon sequential method. The initial loading dose would be increased in the next patient as positive reactions were observed in the patient during endoscopy; otherwise, the initial loading dose would be reduced. In the event of a suspicious effect of sedation such as obvious swallowing, the next patient would receive a similar dosage. The formal study started when the dose reduction was altered to increase or vice versa, and the previous case of this patient was considered the first. The experiments were performed sequentially and terminated after seven negative-positive response crossovers (Supplementary Table 1).

Each patient was scored with MOAA/S (Supplementary Table 2)^6^ at 30 s (t1), 1 min (t2), and 2 min (t3) after the injection of an initial loading dose of remimazolam besylate. Patients with a score higher than 2 after 2 minutes would be deemed to have sedation failure and receive remimazolam besylate of 2.5 mg until successful placement of the gastroscope. Otherwise, patients would be considered as effective sedation. Besides, patients without such deep sedation at any time during the gastroscopy would receive additional remimazolam besylate of 2.5 mg until the gastroscopy was completed, which would be no more than five times within 15 minutes, with a maximum bolus of 19.5 mg. Timely measures would be taken for the treatment of any adverse events during the study.

### Data collection

The initial loading dose of remimazolam besylate, use of the first remedial sedation, MOAA/S scores at t1, t2, and t3, the induction time of sedation, the anesthesia recovery time, the observation time in the resuscitation room, and the adverse reactions were recorded. MAP, HR, and SPO_2_ after entering the operating room (T0), endoscope placement (T1), end of gastroscopy (T2) and when patients left the resuscitation room (T3) were also collected.

### Statistics analysis

SPSS 26.0 statistical software (IBM, Armonk, NY, USA) was used. The Probit regression model was used to calculate ED_50_/ED_95_ and 95% Confidence Interval (95% CI). Data presentation and statistical tests were performed as reported. A two-sided *p* < 0.05 was considered as statistically significant.

## Results

A total of 31 patients were eventually included including 13 (41.9%) males and 18 (58.1%) females. Age and body mass index of all participants were 43.7 ± 12.2 years old and 23.4 ± 3.3 kg.m^−2^, respectively. Eight (25.8%), 15 (48.4%), and 8 (25.8%) patients were classified as ASA I, II or III.

Twenty nine patients’ vital signs were within acceptable limits during gastroscopy. The induction time after injection of remimazolam besylate was 70 ± 25 s, with anesthesia recovery time and observation time in the resuscitation room 5.1 ± 2.1 min and 2.3 ± 1.6 min, respectively.

**Figure 1:**
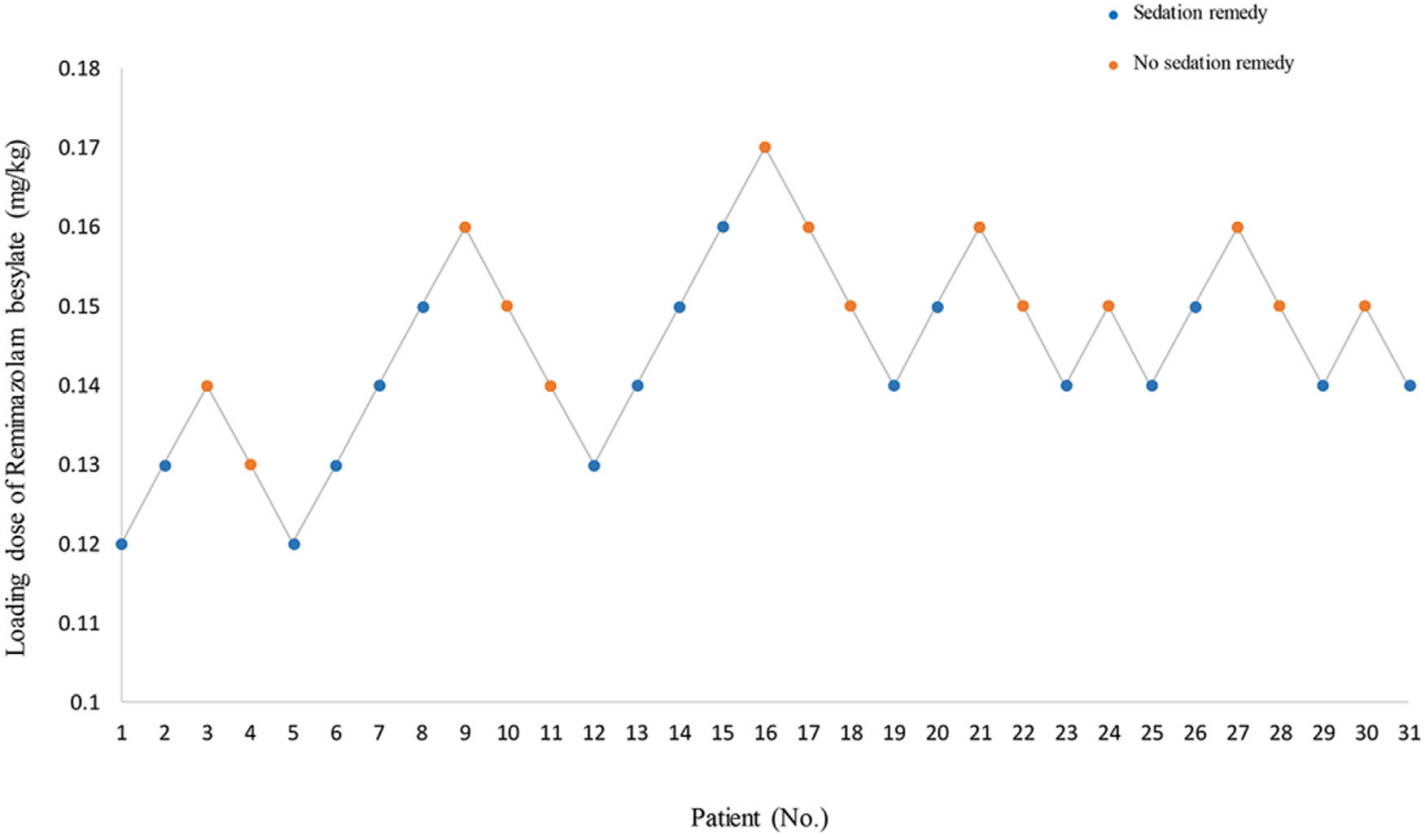
Sequential Experiment Chart. Sequential anesthesia results of remimazolam in anesthesia for gastroscopy.

**Figure 2:**
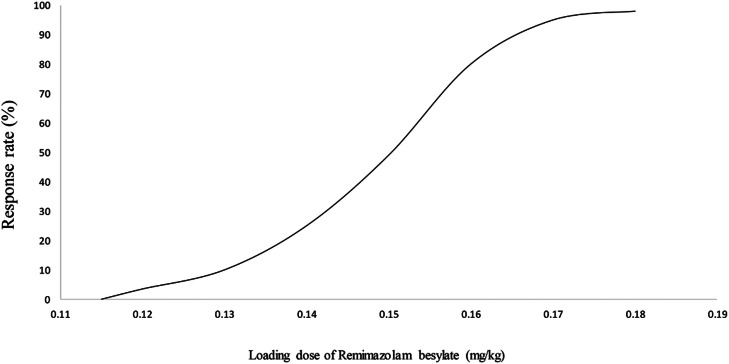
Dose-response curve. Dose-response relationship of remimazolam in anesthesia for gastroscopy.

ED_50_ of the initial loading dose of remimazolam besylate combined with alfentanil was 0.147 mg.kg^−1^ (95% CI 0.138‒0.160 mg.kg^−1^), and ED_95_ was 0.171 mg.kg^−1^ (95% CI 0.159‒0.245 mg.kg^−1^). Fourteen patients had effective sedation and successful gastroscope placement after the initial loading dose administration, and 5 and 12 patients succeeded with an additional 2.5 mg due to sedation failure and positive reactions, respectively (Fig. 1). With the increase in the dosage, the depth of sedation increased, while the positive gastroscopic reactions decreased (Fig. 2).

No significant changes in vital signs were seen except for MAP, which fell significantly at T1 and T2 (both *p* < 0.05 vs. T0) and rose significantly at T3 (*p* < 0.05 vs. T2).

## Discussion

This study showed that the ED_50_ of the initial loading dose of remimazolam besylate combined with alfentanil was 0.147 mg.kg^−1^ and the ED_95_ was 0.171 mg.kg^−1^, which could assist clinical practice of remimazolam besylate during painless gastroscopy.

Endoscopic sedation is widely used in routine practice to relieve anxiety and discomfort, resulting in a successful procedure, and this is also the reason why we focused on this population.^7^^,^^8^ Remimazolam besylate is a new type of benzodiazepine and ultra-short-acting γ-Aminobutyric Acid type A (GABAA) receptor agonist characterized by quick onset, short half-life, thorough removal, quick loss of sedation effect and fast recovery. It is metabolized into inactive products by tissue esterase and excreted through the kidney.^9^ Alfentanil, a new type of opiate, has been widely used for anesthesia in short operations and outside operating rooms for its characteristics of quick onset, high antalgic intensity, short maintenance time, quick metabolism, elevated safety, and awakening quality. Yang et al found that, compared to propofol, remimazolam besylate might have reduced the incidence of delirium in patients who underwent cardiac surgery. In this study, patients were given remimazolam besylate combined with alfentanil before the gastroscopy. We found that the vital signs of most patients were within acceptable limits during gastroscopy except for one who developed hypotension, and one with obvious respiratory depression. The Dixon sequential method is the method most widely used in studies on the dose of anesthetic drugs and has the advantage of saving samples.^10^ Therefore, it was used to determine the initial effective dose of remimazolam besylate for painless gastroscopy in this study. Gradient increase or decrease of remimazolam besylate provides an accurate indication for ED_50_. We further found that the ED_50_ of remimazolam besylate combined with alfentanil for painless gastroscopy was 0.147 mg.kg^−1^, and the ED_95_ was 0.171 mg.kg^−1^.

This study had some limitations. The study was a single-centered study. Besides, no serum assays of remimazolam besylate and alfentanil were detected.

## Conclusions

In conclusion, the ED_50_ of remimazolam besylate combined with alfentanil for adult gastroscopy was 0.147 mg.kg^−1^, and the ED_95_ was 0.171 mg.kg^−1^.

## Ethics approval statement

This study was approved by the Ethics Committee of Anhui nº 2 Provincial People's Hospital.

## Patient consent statement

All patients had voluntarily signed the informed consent.

## Funding

None.

## Declaration of competing interest

The authors declare no conflicts of interest.

## Data Availability

The data set supporting the results of this article are included within the article.

## Appendix Supplementary materials

Image, application 1

## Footnotes

Supplementary material associated with this article can be found in the online version at doi:10.1016/j.bjane.2024.844518.
