# Supplementary material for: Determination of ED50 and ED95 of remimazolam besylate combined with alfentanil for adult gastroscopy: a prospective dose-finding study
Source: Braz J Anesthesiol. 2024 May 23;74(4):844518. doi: 10.1016/j.bjane.2024.844518 (PMC11167255; doi:10.1016/j.bjane.2024.844518)
Supplement: Supplementary file 1 [file mmc1.docx]

BJAN-D-23-00394_Supplementary Material

**Supplementary Table 1** Steward Awake score scale used in this study.

| **Patient condition** | **Score** |
| --- | --- |
| **1. Awakening degree** |  |
| Fully awake | 2 |
| Responses to stimuli | 1 |
| No response to stimulation | 0 |
| **2. Respiratory tract patency degree** |  |
| Cough according to doctor's instructions | 2 |
| Able to independently maintain airway patency | 1 |
| Respiratory tract needs support | 0 |
| **3. Degree of limb activity** |  |
| Limbs can do conscious activities | 2 |
| Unconscious limb activity | 1 |
| Limb inactivity | 0 |

**Supplementary Table 2** Modified Observer's Alertness/Sedation scale (MOAA/S) used in this study.

| **Score** | **Standard** |
| --- | --- |
| 5 | Quick response to normal voice calls |
| 4 | Inresponse to normal voice calls |
| 3 | Call out or call repeatedly, eyes open |
| 2 | Slightly shaking the body, such as the head or shoulders, causing reactions |
| 1 | Response to nociceptive stimuli |
| 0 | Non-responsive to nociceptive stimuli |
